# Genotypic Variants of Pandemic H1N1 Influenza A Viruses Isolated from Severe Acute Respiratory Infections in Ukraine during the 2015/16 Influenza Season

**DOI:** 10.3390/v13112125

**Published:** 2021-10-21

**Authors:** Oksana Zolotarova, Anna Fesenko, Olga Holubka, Larysa Radchenko, Eric Bortz, Iryna Budzanivska, Alla Mironenko

**Affiliations:** 1 Educational Scientific Centre “Institute of Biology and Medicine”, Taras Shevchenko National University of Kyiv, 01601 Kyiv, Ukraine; birishechka68@gmail.com; 2 Gromashevsky L.V. Institute of Epidemiology and Infectious Diseases, National Academy of Medical Sciences of Ukraine, 03680 Kyiv, Ukraine; fesna2007@ukr.net (A.F.); olg_holubka@ukr.net (O.H.); larysa_rad@ukr.net (L.R.); miralla@ukr.net (A.M.); 3 Department of Biological Sciences, University of Alaska, 3211 Providence Dr., Anchorage, AK 99508, USA; ebortz@alaska.edu

**Keywords:** influenza, H1N1, A(H1N1)pdm09, phylogenetics, acute respiratory infection, mutation

## Abstract

Human type A influenza viruses A(H1N1)pdm09 have caused seasonal epidemics of influenza since the 2009–2010 pandemic. A(H1N1)pdm09 viruses had a leading role in the severe epidemic season of 2015/16 in the Northern Hemisphere and caused a high incidence of acute respiratory infection (ARI) in Ukraine. Serious complications of influenza-associated severe ARI (SARI) were observed in the very young and individuals at increased risk, and 391 fatal cases occurred in the 2015/16 epidemic season. We analyzed the genetic changes in the genomes of A(H1N1)pdm09 influenza viruses isolated from SARI cases in Ukraine during the 2015/16 season. The viral hemagglutinin (HA) fell in H1 group 6B.1 for all but four isolates, with known mutations affecting glycosylation, the Sa antigenic site (S162N in all 6B.1 isolates), or virulence (D222G/N in two isolates). Other mutations occurred in antigenic site Ca (A141P and S236P), and a subgroup of four strains were in group 6B.2, with potential alterations to antigenicity in A(H1N1)pdm09 viruses circulating in 2015/16 in Ukraine. A cluster of Ukrainian isolates exhibited novel D2E and N48S mutations in the RNA binding domain, and E125D in the effector domain, of immune evasion nonstructural protein 1 (NS1). The diverse spectrum of amino-acid substitutions in HA, NS1, and other viral proteins including nucleoprotein (NP) and the polymerase complex suggested the concurrent circulation of multiple lineages of A(H1N1)pdm09 influenza viruses in the human population in Ukraine, a country with low vaccination coverage, complicating public health measures against influenza.

## 1. Introduction

Human influenza A viruses (IAV) are a major threat to public health and the global economy, causing annual seasonal epidemics and occasional novel pandemics. Influenza viruses, including the H1N1 swine-origin 2009 pandemic lineage viruses, H3N2 viruses, and influenza B viruses, infect 5–10% of adults and 20–30% of children annually [1]. The original H1N1 2009 pandemic virus is referred to as pdmH1N1, while the lineages of seasonal descendants of the pandemic strain are referred to as A(H1N1)pdm09 [1]. Importantly, influenza viruses continually evolve as a result of immune pressure (antigenic drift), often resulting in altered pathogenicity, and changing epidemiological patterns to produce immune escape mutants that can affect the annual vaccine antigen selection and efficacy of human influenza vaccines [2].

When effective, influenza A and B vaccines protect the general population and particularly people from high-risk groups (young children, pregnant women, health care workers, and the elderly) against severe disease, or influenza-like illness (ILI). However, the vaccine composition must be annually changed because of antigenic drift among influenza viruses in the human population.

The development of recommendations for vaccine composition is based on phylogenetic and immunological analyses of influenza viruses in the previous epidemic season. The phylogenetic analysis is, however, usually limited just to the analysis of nucleotide gene sequences of viral hemagglutinin (HA) and neuraminidase (NA) genes. Even using state-of-the-art analysis methods, including antigenic cartography built from hemagglutination inhibition (HI) assay data [3], the prediction of antigenicity and antibody responses to seasonal variants of influenza A and B viruses has been problematic [4]. New studies increasingly use an analysis of the entire genome of influenza viruses that can shed new light on the evolution of viruses and immune pressures that may be useful for selecting vaccine strains for future use in subsequent seasons [5].

Type A influenza viruses, particularly pdmH1N1 viruses and their genetic descendants that are the cause of seasonal H1N1 epidemics, continually evolve in the human population. H1N1 viruses have accumulated mutations that are associated with higher replication (fitness), increased binding to α-2,6-sialic acid receptors on respiratory epithelial cells, and escape from innate and adaptive immune responses [6]. H1N1 viruses were a leading cause of influenza-like illness (ILI) over the 2015/16 epidemic season. In Taiwan, for example, hemagglutinin (HA) clade 6B/6B.1/6B.2 infections were associated with an increased severity of influenza in hospitalized patients, including a higher risk of pneumonia and acute respiratory distress syndrome (ARDS), and complications requiring mechanical ventilation [7]. In the 2015/16 epidemic season in Ukraine, influenza viruses were the cause of a large number of hospital admissions for severe acute respiratory infection (SARI) and serious respiratory complications of both young people and people at risk [8], resulting in 391 fatal cases. To understand the genetic basis for the severity of ILI and SARI in Ukraine, we conducted molecular genetics and phylogenetics analyses of A(H1N1)pdm09 influenza viruses from SARI cases that circulated in Ukraine during the 2015/16 epidemic season. We found that most of the A(H1N1)pdm09 influenza viruses circulating in Ukraine were of the H1 6B.1 lineage that became predominant worldwide and was associated with antigenic escape from immunity [7,8,9,10]. Our study also revealed the emergence of new genotypic variants in Ukraine, containing unique amino-acid substitutions in HA, NS1, and other viral genes.

## 2. Results

### 2.1. Epidemiology of Seasonal Influenza in Ukraine, 2015/16

**Figure 1:**
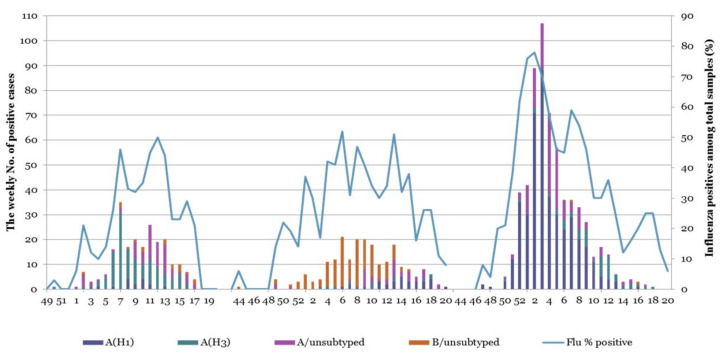
Weekly confirmed cases of influenza in the 2013/14, 2014/15, and 2015/16 seasons in Ukraine among clinical surveillance for ILI in four cities in Ukraine (Kyiv, Dnipro, Odessa, and Khmelnytsky). Influenza type and subtype were determined by RT-PCR and antigenic testing from nasal swabs/sputum.

In Ukraine during the 2015/16 influenza epidemic season, it was hypothesized that the A(H1N1)pdm09 influenza strains that were circulating in the Northern Hemisphere were contributing to markedly increased disease incidence and serious complications in patients. The A(H1N1)pdm09 influenza subtype dominated the epidemic curve in the 2015/16 season (Figure 1). While 391 persons were reported as having died in Ukraine from influenza and its complications, the subsequent rapid decline of cases suggests that the epidemic threshold was not exceeded (based on all flu cases in population) for a sustained period.

Influenza vaccination rates in Ukraine during 2015/16 were only 0.3% in comparison to those in the European Union (EU), due to a lack of systematic national or regional-level vaccination campaigns [11]. However, much of the population may have possessed some degree of immunity to previous A(H1N1)pdm09 influenza lineages through natural exposure to IAV circulating in the human population since the 2009 pandemic. While there could be underlying causes that explain the rapid rise of SARI incidence in 2015/16, for example, the late seeking of medical care or predisposing obesity or chronic diseases, it is also known that waning immunity, or a change in virus genotypes resulting in escape from pre-existing immunity, is a common driver of severe seasonal epidemic years in influenza [11]. In aggregate, approximately 25% of 4964 SARI patients we identified from ten sentinel hospitals in four cities in Ukraine (Kyiv, Dnipro, Odessa, and Khmelnytsky) in the 2015/16 influenza season suffered from comorbidities (asthma, obesity, diabetes, or cardiovascular and/or lung diseases). We sampled a random subset of 1246 hospitalized SARI cases (25% of all SARI patients) and tested these cases by PCR. Of these, 341 were positive for influenza A or B, with 335 (98%) of these cultured and identified as the A(H1N1)pdm09 influenza strain.

In order to improve our understanding of the Ukrainian 2015/16 epidemic, we analyzed the genetic changes that occurred in the dominant influenza subtype, A(H1N1)pdm09, during this period. While in virus genetics studies of IAV, the focus is typically directed to the surface proteins (HA and NA) that are the key targets for neutralizing antibodies, changes in the virulence and pathogenicity of influenza viruses can also be harbored mutations in the polymerase, matrix and nucleoprotein, and non-structural proteins [12]. Thus, we analyzed the phylogeny and amino acid sequences of all genome segments of 73 type A(H1N1)pdm09 influenza viruses from Ukraine isolated in the 2015/16 epidemic (strains listed in Table S1).

### 2.2. Phylogenetic Analysis of HA Protein

Viruses isolated in Ukraine in the 2015/16 season belong to the 6B genetic group in which two new subgroups, namely 6B.1 and 6B.2, emerged in that season [7]. These subgroups are characterized by specific aminoacyl substitutions. According to results of the phylogenetic analysis of H1 HA (gene segment 4), it was found that all Ukrainian isolates of the 2015/16 season were genetically clustered with the A/California/07/2009 (H1N1) strain that forms the basis of current influenza vaccines. Interestingly, in the 2015/16 season, the pandemic influenza viruses caused a large number of hospital admissions for serious complications of both young people and people with underlying risk factors [13].

**Figure 2:**
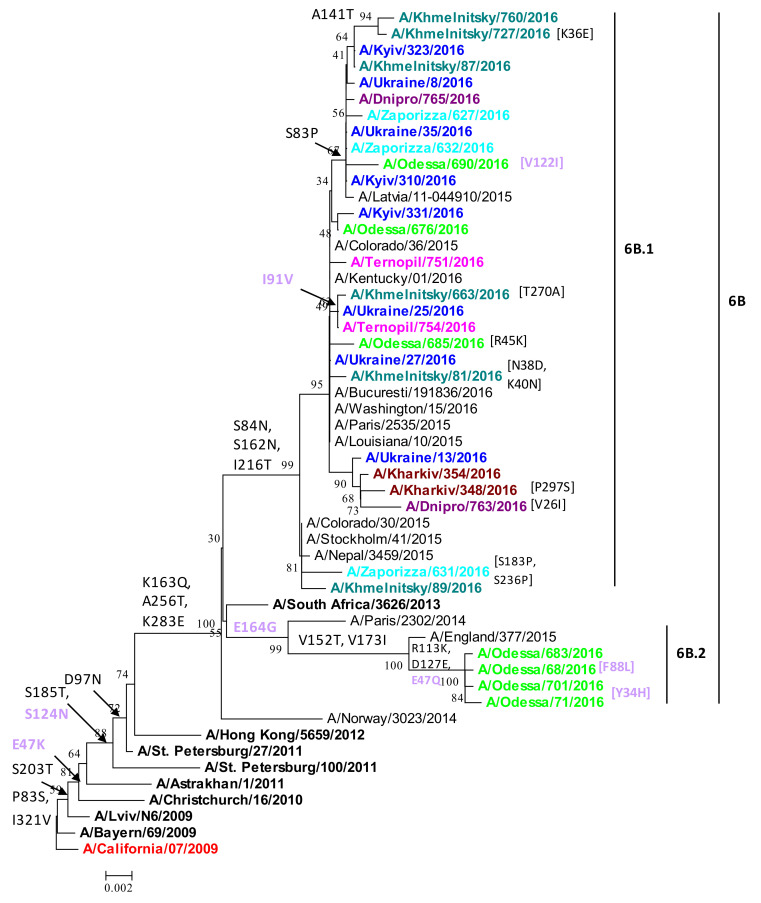
Phylogenetic analysis of A(H1N1)pdm09 influenza virus hemagglutinin (HA) nucleotide sequences from the 2015/16 season in Ukraine. Phylogenetic trees were constructed by the NJ (neighbor-joining) method, and Kimura 2-parameter model, with 1000 bootstrap replications. Amino acid variations are labeled for 6B.1 and 6B.2 subgroups.

The phylogenetic analysis of HA revealed a number of amino-acid substitutions in A(H1N1)pdm09 influenza viruses from Ukraine isolated during the 2015/16 epidemic. HA P83S, I321V, and S203T substitutions existed in Ukrainian isolates (Figure 2). These substitutions were also observed in isolates of the previous epidemic season (2014/15), unlike the exception of the pdmH1N1(CA04) and the A/Bayern/69/2009 vaccine strains. All isolates, except for the reference strains, also had the amino-acid substitutions such as D97N, S185T, E47K (HA2), and S124N (HA2). These amino-acid substitutions were fixed over previous influenza seasons.

In the large Ukrainian 6B clade branching off the A/Norway/3023/2014 strain, the K163Q, A256T, K283E substitutions also arose. Such mutations are characteristic of the 6B genetic group, which is divided into two subgroups, 6B.1 and 6B.2 (Figure 2). Most of the isolates from Ukraine belonged to a new genetic group (6B.1). This group had specific mutations such as S84N, S162N (forming a potential glycosylation site), and I216T. Three isolates from Khmelnitsky, Kyiv, and Ternopil formed a separate group having acquired the I91V (HA2) substitution. Some Ukrainian isolates together with an isolate from Latvia were reversed in position 83 to proline.

Four Ukrainian isolates from Odessa were in the 6B.2 subgroup, having acquired HA substitutions R113K, D127E (a potential glycosylation site), E164G (HA2), and the E47Q (HA2). Similar to the A/England/377/2015 strain, they also harbored substitutions characteristic of the 6B.2 genetic group at V152T and V173I. The rare V152T aminoacyl substitution has occurred in 18 isolates in nature, and in cell culture, and affects antigenic properties [1].

**Figure 3:**
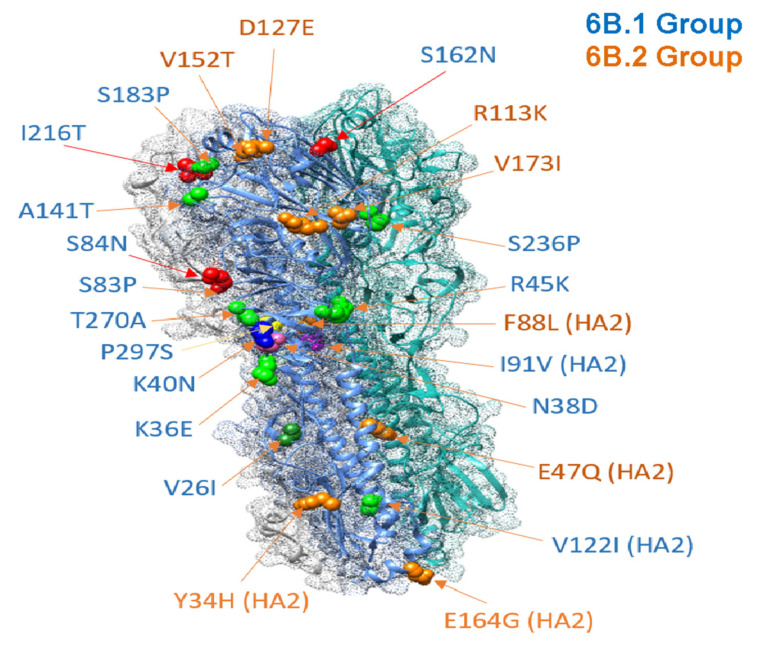
Model 3D structure of the A(H1N1)pdm09 HA protein with the substitutions that occurred in 2015/16 in strains isolated in Ukraine. Structure (PDB ID—3LZG) was modeled using Chimera software and labeled according to H1 6B.1 (blue) or 6B.2 (orange) genetic group.

*Variation of potential glycosylation sites in HA.* The acquisition or loss of glycosylation sites leads to changes in the topology of the hemagglutinin (HA) protein structure. Acquiring a site for glycosylation may be beneficial for the virus, as, due to this, the masking of antigenic sites that are recognized by the antibodies of the human organism occurs. Thus, the viruses can potentially regulate their antigenicity [14]. For A(H1N1)pdm09 influenza viruses in the 2015/16 season, there were two mutations, namely S162N and D127E, which may lead to glycosylation site acquisition. Three amino-acid substitutions were common for all viruses belonging to the 6B.1 genetic group in Ukraine—the S84N, S162N, and I216T, suggesting variation in glycosylation patterns (Figure 3).

In the 2015/16 epidemic season, there were also a large number of unique amino-acid substitutions that were not observed in the previous epidemic seasons (Figure 3).

**Figure 4:**
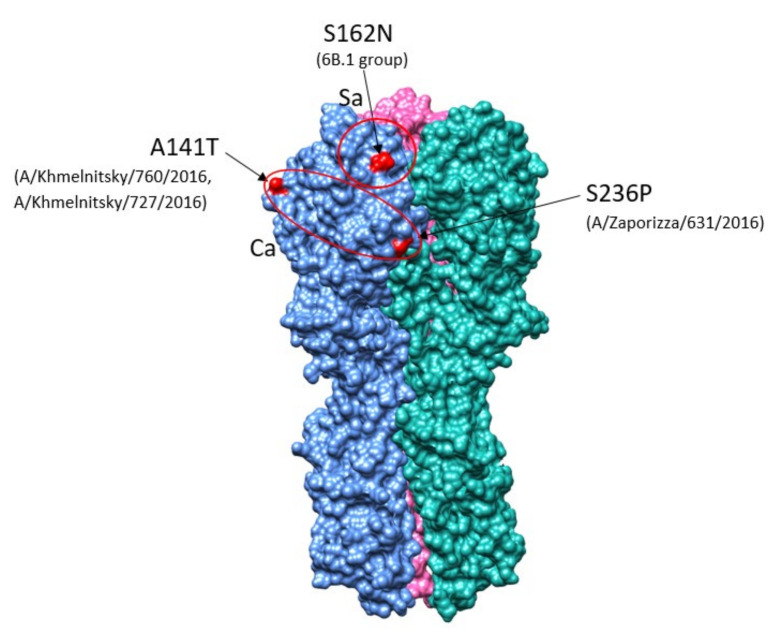
3D structure of HA molecule with changes in antigenic sites of Ukrainian isolates (PDB ID—3LZG).

*Antigenic site variations in HA.* It is known that the H1 molecule of hemagglutinin has four antigenic sites, namely Sa, Sb, Ca, and Cb [15]. These sites consist of the most variable amino acids and are exposed to the immune system antibodies. It should be noted that Sa and Sb sites, which contain many amino acids involved in neutralizing epitopes, are located near the receptor pocket [15]. Ukrainian isolates had substitutions in antigenic sites that emerged in the 2015/16 season and were not detected previously. The S162N main substitution emerged in the Sa antigenic site and was observed in all isolates of the 6B.1 genetic group [16] (Figure 4).

Two substitutions were found in the Ca antigenic site. The A141T substitution was acquired by 2016 isolates No. 727 and No. 760 from Khmelnitsky, and S236P by the A/Zaporizza/631/2016 isolate. The information on changes in antigenic sites is very important for predicting the following dominant strains. It is well documented that antigenic changes lead to the acquisition of hydrocarbon side chains on the HA molecule [17]. As the hydrocarbon chains are located near antigenic sites, they mask the neutralizing epitopes on the surface of HA. Antigenic substitutions that lead to the acquisition of glycosylation sites are believed to generate new antigenic variants effectively.

A number of other unique amino-acid substitutions in Ukrainian isolates were also identified (Table 1). Together with genetic changes described in the 6B.1 subclade, these genetic variations may have provided A(H1N1)pdm09 influenza viruses with new antigenic properties, including glycosylation sites that can facilitate escape from previously existing antibody responses, whether memory was generated by a naturally acquired infection or vaccination. The A(H1N1)pdm09 influenza 6B.1 group has since become widespread [1,13,18].

### 2.3. Phylogenetic Analysis of NA Protein

**Figure 5:**
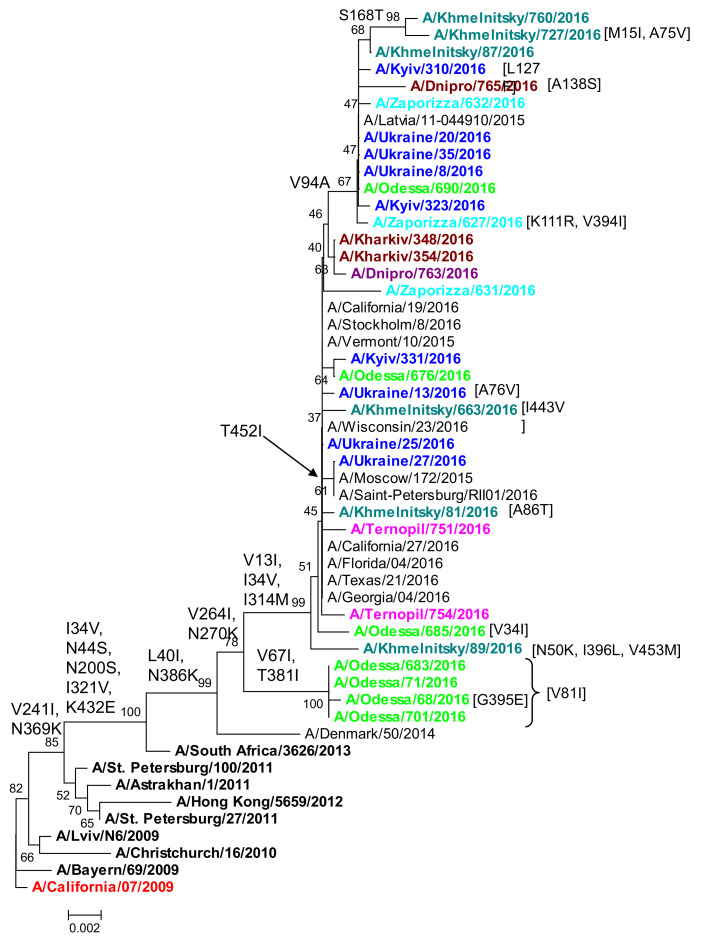
Phylogenetic comparison of A(H1N1)pdm09 influenza virus NA from the 2015/16 season. NA nucleotide sequences were used to build a tree by NJ method, Kimura 2-parameter model, with 1000 bootstrap replications. Amino acid variations are indicated; strains from Ukraine are colored; reference strains are black, and the vaccine strain is red.

From our analysis of neuraminidase (gene segment 6) sequences, we found that all A(H1N1)pdm09 isolates sequenced from the 2015/16 season in Ukraine formed a subclade of the A/SouthAfrica/3626/2013 reference strain [13]. Moreover, the NA phylogenetic tree phylocopied the HA gene tree (Figure 2) with a parallel division of influenza viruses into the two 6B.1 and 6B.2 subgroups (Figure 5). Overall, the N1 Na in A(H1N1)pdm09 influenza viruses isolated during the 2015/16 season in Ukraine were genetically similar (on average 98.8%) to the vaccine strain.

All Ukrainian isolates in the clades phylogenetically descending from the common ancestor of A/St.Petersburg/27/2011 and A/SouthAfrica/3626/2013 acquired the amino-acid substitutions V241I and N369K. The A/SouthAfrica/3626/2013 subclade, that included all Ukrainian isolates, harbored I34V, N44S, N200S, I321V, and K432E, substitutions that were absent in most of the reference strains (Figure 5). The majority of isolates also had the L40I and N386K substitutions.

Most of the Ukrainian isolates belonged to the 6B.1group and acquired amino-acid substitutions such as V13I, I34V, V264I, N270K, and I314M (Figure 5). The A/Khmelnitsky/89/2016 isolate also had N50K, I396L, and V453M. Virus isolate No. 685 isolated in Odessa acquired the V34I point substitution. Three isolates had a T452I mutation, and two 2016 isolates (No.81 and No.663) from Khmelnitsky had A86T and I443V point substitutions, respectively. The isolate from Zaporizhia had two unique substitutions (K111R and V394I). A subgroup of 2016 Ukrainian isolates in a subclade that included A/Latvia/11-044910/2015 had V94A and the unique point substitutions: A/Dnipro/765/2016 had A138S; A/Kyiv/310/2016 had L127F; A/Khmelnitsky/87/2016 had M15I and A75V; and A/Khmelnitsky/760/2016 had S168T. The H1 6B.2 group of isolates from Odessa (Figure 2) also acquired V67I, T381I, and V81I in NA; the A/Odessa/68/2016 isolate acquired the unique G395E point substitution. Substitutions associated with the emergence of resistance to oseltamivir and other next-generation antiviral drugs were absent. However, the plethora of unique amino acid variations in NA, similar to HA, suggest that immune pressure (antibody responses) may have driven antigenic drift in human A(H1N1)pdm09 infections in Ukraine.

### 2.4. Phylogenetic Analyses of Internal Genes: M1, M2, and NP

**Figure 6:**
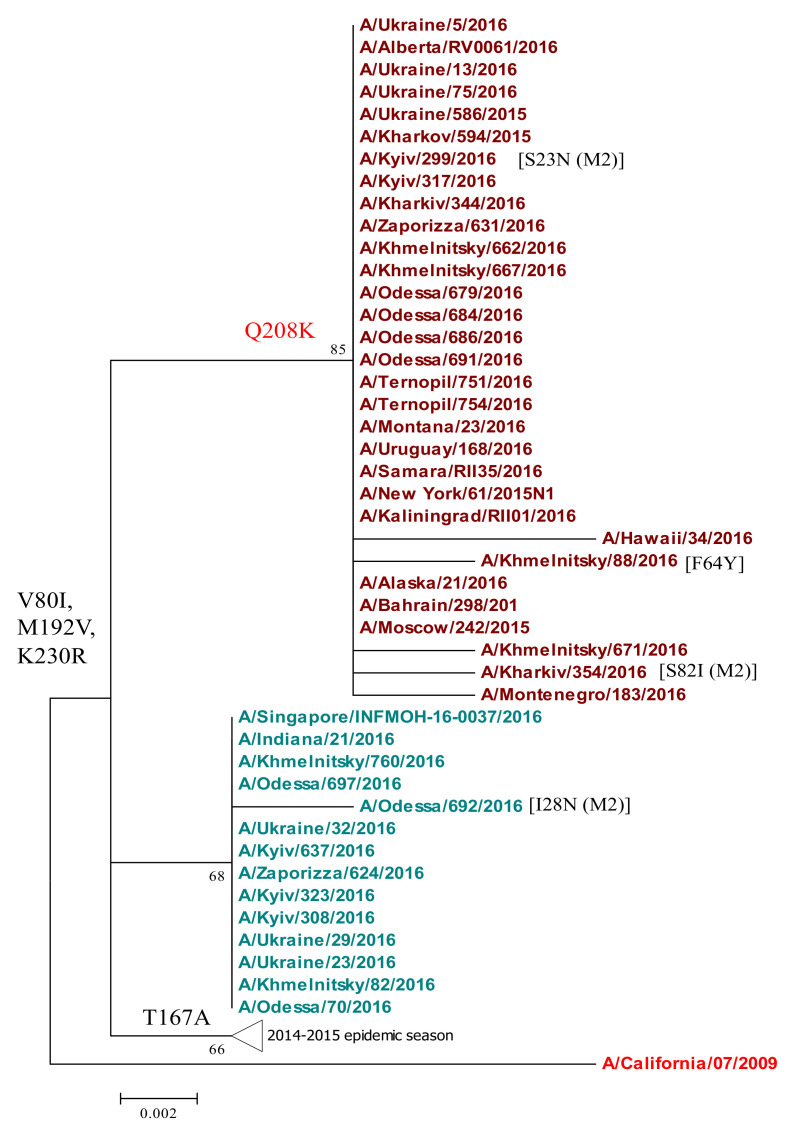
Phylogenetic analysis of A(H1N1)pdm09 influenza virus M1 from the 2015/16 season. M1 gene nucleotide sequences were analyzed by NJ method, Kimura 2-parameter model, with 1000 bootstrap replications. Ukrainian clades are colored except the vaccine strain which is red; amino acid variations are indicated as arising at specific branch nodes; M2 substitutions are also annotated (M2).

*Matrix proteins.* Two matrix proteins, M1 and M2, are encoded in the MP gene (segment 7) of influenza A viruses. For phylogenetic analysis, isolates were taken from the 2014/15 and 2015/16 epidemic seasons. All isolates including the A/California/07/2009 vaccine strain had the D21G mutation in the M2 protein (Figure 6), which causes resistance to antiviral drugs amantadine and rimantadine, which are ion channel inhibitors. A few isolates also contained unique point substitutions in the M2 protein (S23N, I28V, and S82I).

*M1 protein.* Isolates of the 2014/15 and 2015/16 epidemic seasons in Ukraine acquired three amino-acid substitutions in the M1 protein, V80I, M192V, and K230R, which differed from the vaccine strain. However, the viruses divided into three genetic groups in the M1 phylogenetic tree (Figure 6). Isolates isolated during the 2014/15 season had the T167A substitution, which was not fixed the next season. The 2015/16 isolates formed two more subclades, one of which acquired the new Q208K substitution in the M1 protein (Figure 6). For the first time among viruses sequenced in 2015/16, the Q208K substitution, that was initially discovered in 2013 and in 2015 in Ukraine, was observed in 62.5% of isolates of the 2015/16 epidemic season.

**Figure 7:**
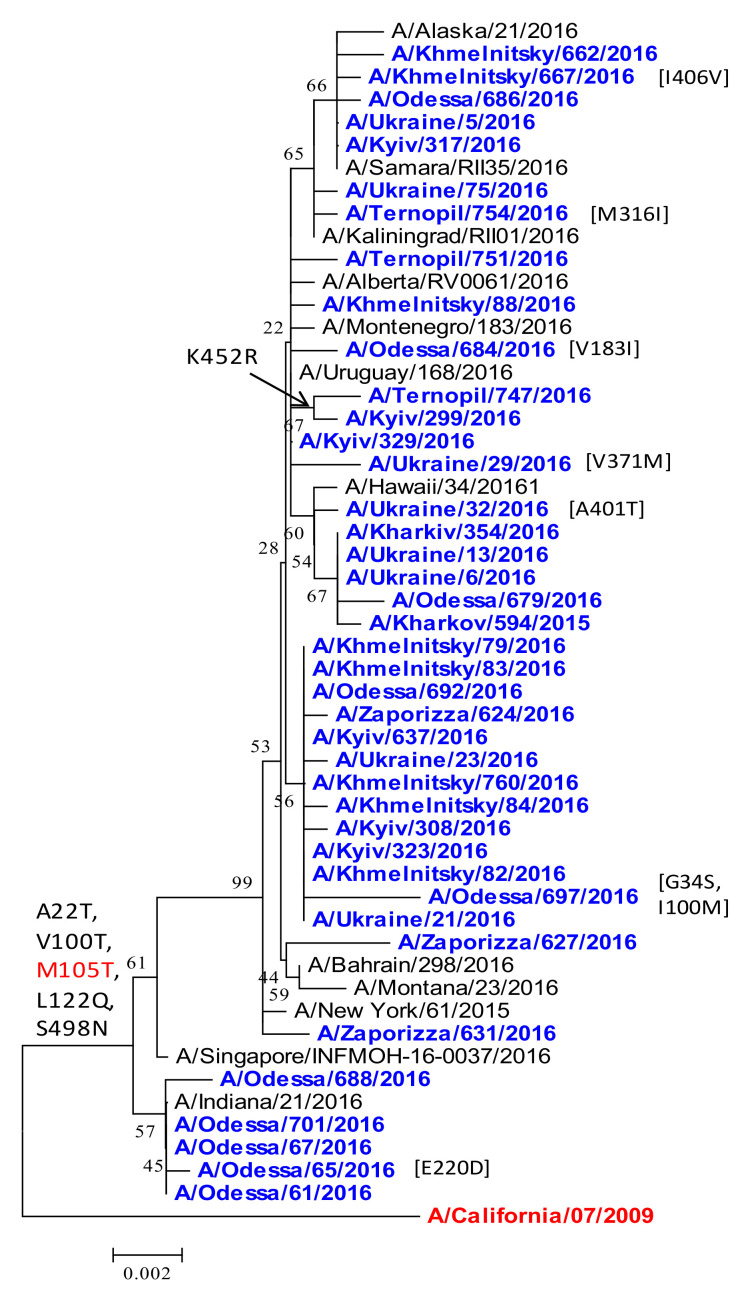
Phylogenetic comparison of A(H1N1)pdm09 influenza virus NP from the 2015/16 season. NP nucleotide sequences were analyzed by NJ method, Kimura 2-parameter model, with 1000 bootstrap replications. Ukrainian clades are colored blue; reference strains are black, except the vaccine strain (red/bold); amino acid variations are indicated, with M195T common in all isolates (red).

*NP.* There was comparatively less amino acid variation in A(H1N1)pdm09 influenza virus NP for the 2015/16 season in Ukraine. However, isolates differed from the vaccine strain with the A22T, V100T, L122Q, S498N substitutions (Figure 7). These new amino-acid substitutions were not detected among earlier Ukrainian isolates. The M105T substitution was found in all of the studied isolates, and was characteristic not only for Ukrainian viruses, but also for viruses from other countries. According to the EpiFlu GISAID data, the spread of the M105T substitution in influenza strains around the world increased from 6% in the 2013/14 season to 86% in the 2015/16 season.

### 2.5. Phylogenetic and Structural Analysis of Non-Structural (NS) Proteins

Two nonstructural proteins, NS1 and NS2, are encoded at the eighth segment of the influenza virus genome. Viral protein NS1 plays a major role in antagonizing cellular processes that restrict viral replication, including the functions of interferon-stimulated antiviral genes (ISG) such as PKR and OAS, modulating pro-inflammatory gene expression, and inducing host protein shutoff by disrupting mRNA processing and export [35].

**Figure 8:**
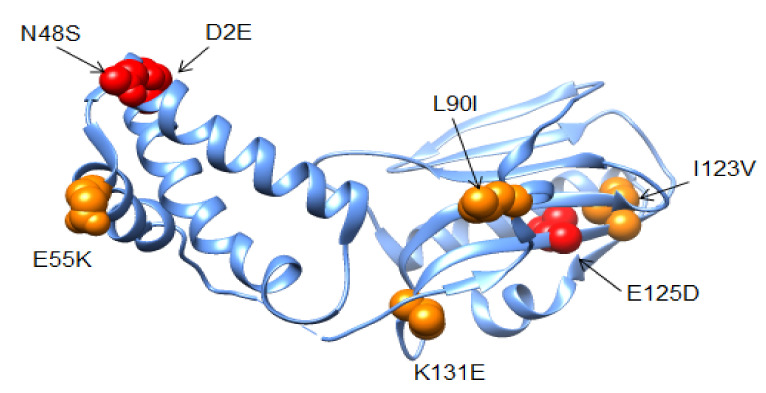
3D structure of NS1 molecule with changes in the Ukrainian isolates (PDB ID—3F5T). New mutations of NS1 of A(H1N1)pdm09 are shown in red. (Chimaera).

Ukrainian isolates differed from the vaccine strain with NS1 substitutions E55K, L90I, I123V, K131E, and N205S, which were common in other viruses in the 2014/15 and 2015/16 epidemic years (Figure 8). Recent studies suggested that NS1 E55K, L90I, I123V, E125D, K131E, and N205S contribute to increased fitness for replication in cultured human lung cells (A549) and occur in combination with mutations in PA and PA-X [12]. This spectrum of NS1 adaptions that occurred in human pdmH1N1 strains in Ukraine was associated also with the restriction of host gene expression, potentially limiting early innate immune responses against influenza [12]. Correspondingly, viruses harboring these NS1 mutations exhibited increased virulence in mouse models of influenza [12].

In the 2015/16 season, a series of new amino-acid substitutions was found in the NS1 protein in the major subgroup of strains in Ukraine, as follows: D2E, N48S, and E125D (Figure 8). All isolates of this subgroup had the D2E and E125D mutations, which were observed in 70% of Ukrainian isolates. According to EpiFlu GISAID data, the outspread of the D2E and E125D substitutions in the NS1 protein of the A(H1N1)pdm09 influenza viruses increased dramatically in the world in less than a year, from 10% in 2015 to 74% in the 2015/16 epidemic season, suggesting that these substitutions confer increased viral fitness [38].

**Figure 9:**
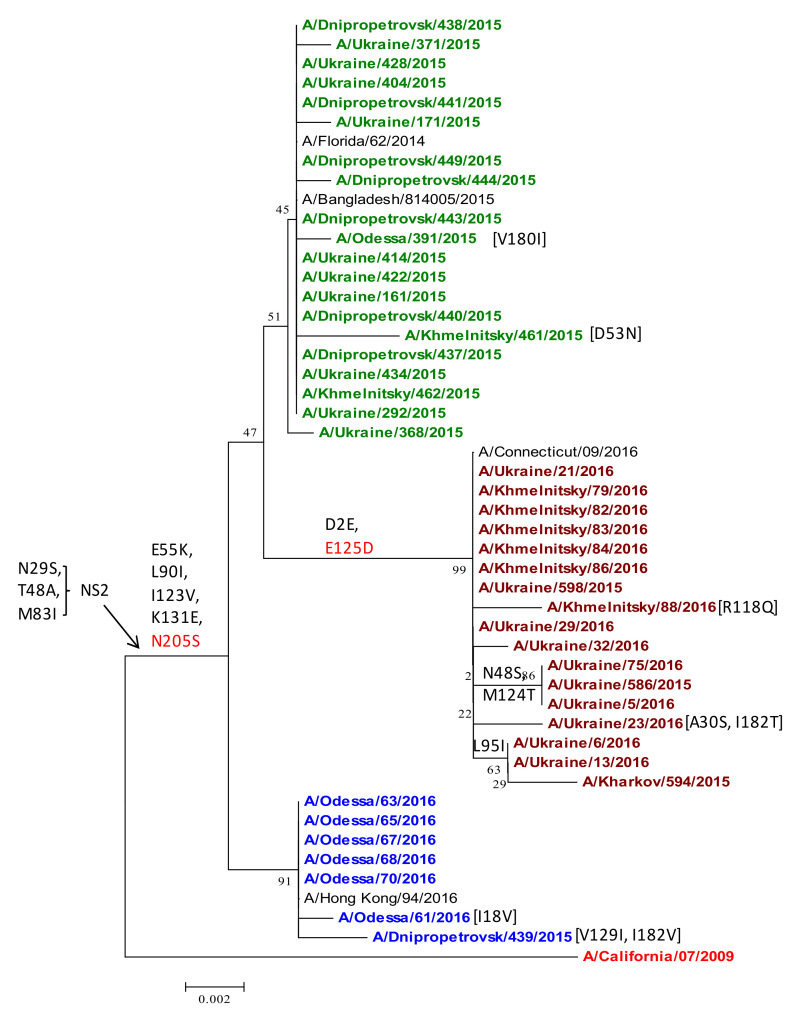
Phylogenetic comparison of A(H1N1)pdm09 influenza virus NS1 from the 2015/16 season. NS1 nucleotide sequences were analyzed by NJ method, Kimura 2-parameter model, with 1000 bootstrap replications. The Ukrainian 2014/15 subgroup (green) was distinct from 2015/16 isolates (brown) and a separate subgroup (blue); reference strains are black, except the vaccine strain (red/bold).

It was suggested that D2E and N48S in the RNA binding domain, and E125D in the NS1 effector domain, are involved in host adaptation (Figure 9). E125D is one of the key substitutions involved in inhibiting the transport of host mRNA and controlling gene expression in human cells. The NS1 protein of all seasonal viruses of (pre-2009 H1N1 and H3N2) influenza contains D125, which interacts with the cleavage and polyadenylation specificity factor 30 (CPSF 30); thus, the E125D mutation may mark host adaptation by recent A(H1N1)pdm09 influenza strains [36].

Some isolates had other substitutions in NS1 such as N48S and M124T, which were identified in 12.5% of the studied viruses; three isolates had the L95I replacement. A second subgroup of seven isolates lacked the mutations of the major subgroup but instead included I18V, or V129I and I182V, respectively (Figure 9).

*NS2.* In the NS2 protein, three amino-acid substitutions, N29S, T49N, and M83I, were found compared to the vaccine strain. However, how these mutations might affect viral virulence is unknown.

### 2.6. Phylogenetic Analysis of Polymerase Complex Genes

The RNA-dependent RNA polymerase complex of influenza viruses is a heterotrimer that consists of three proteins, encoded by three separate gene segments: PA, PB1, and PB2. Besides viral transcription and replication, the polymerase complex is increasingly recognized as an essential host factor for host adaptation [49].

**Figure 10:**
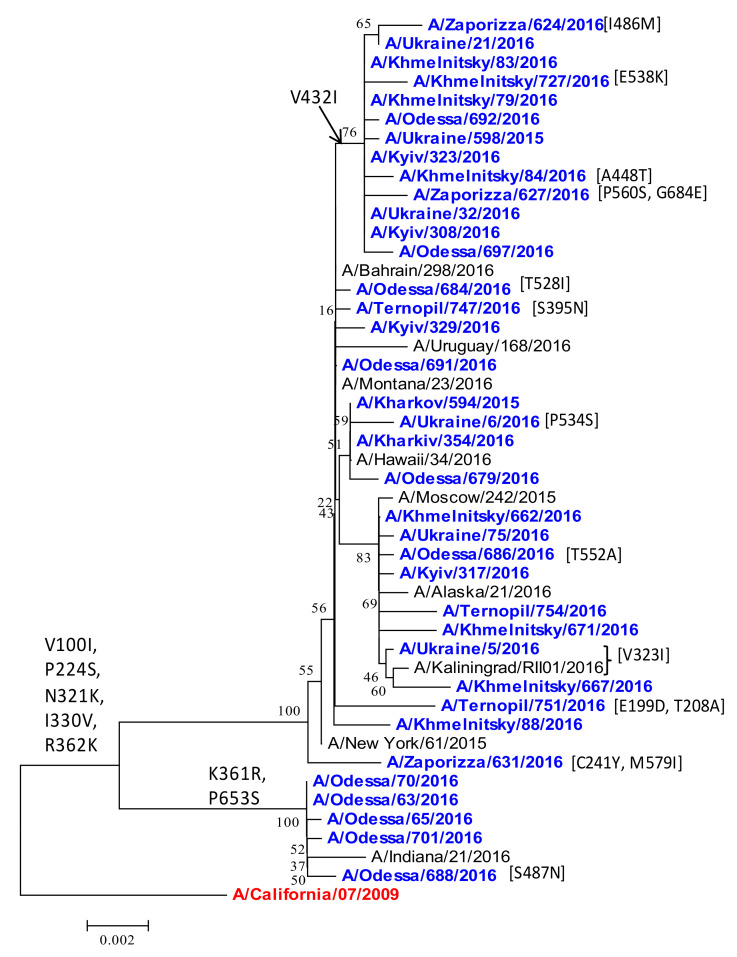
Phylogenetic comparison of A(H1N1)pdm09 influenza virus PA from the 2015/16 season. PA nucleotide sequences were analyzed by NJ method, Kimura 2-parameter model, with 1000 bootstrap replications. The Ukrainian 2015/16 isolates (blue), reference strains (black), and the vaccine strain (red/bold).

*PA polymerase protein.* The PA gene remained similar to the vaccine strain, with a few notable substitutions. All Ukrainian isolates acquired V100I, P224S, N321K, I330V, and R362K (Figure 10). In the 2015/16 season, the P224S mutation spread among the majority of viruses, and it is detected in 99.31% of isolates in GISAID. Recent studies suggested that PA V100I, P224S, N321K, I330V, and R362K contribute to increased fitness for replication in cultured human lung cells (A549), and occur in combination with mutations in NS1, and in the PA-X protein that is generated from an alternate (+1) open-reading frame during the translation of viral mRNA from segment 3 (PA) of the IAV genome [12]. The PA N321K mutation is also interesting and may be indicative of swine-origin virus adaptation to the human population, causing a transition from the severe epidemic to the mild epidemic mode [6]. PA N321K increases viral polymerase activity in vitro and viral replication in cell culture [6,9]. Several other substitutions were noted; I330V was detected in 27.22% of PA in GISAID data and in all Ukrainian isolates. Five isolates from Odessa formed a separate group, having acquired K361R and P653S. Other isolates acquired unique point amino-acid substitutions.

**Figure 11:**
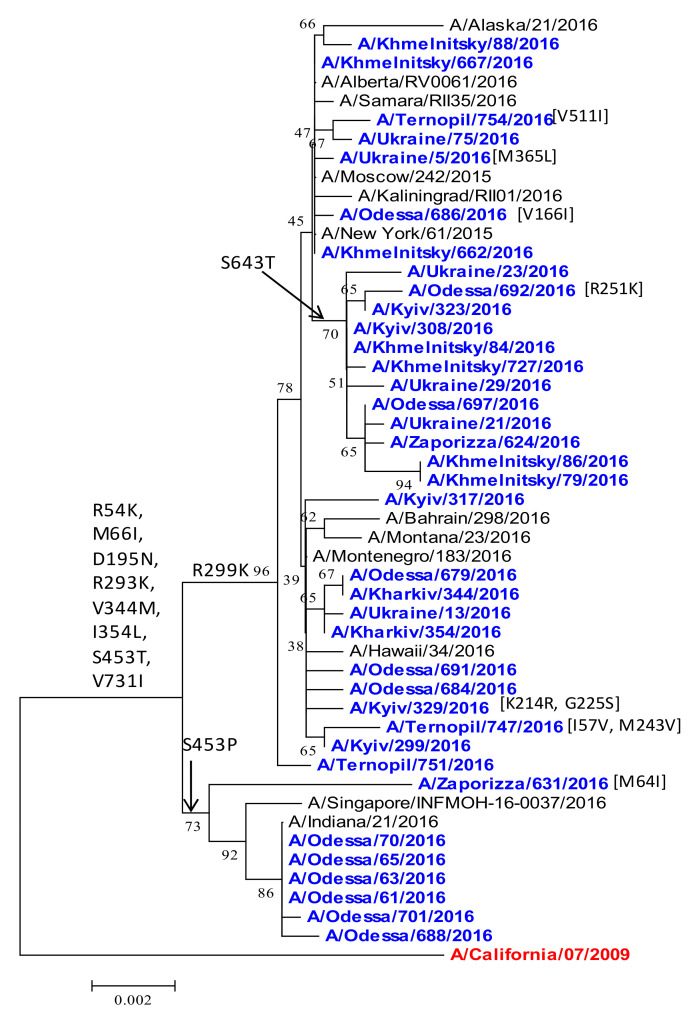
Phylogenetic comparison of A(H1N1)pdm09 influenza virus PB2 from the 2015/16 season. PB2 nucleotide sequences were analyzed by NJ method, Kimura 2-parameter model, with 1000 bootstrap replications. The Ukrainian 2015/16 isolates (blue), reference strains (black), and the vaccine strain (red/bold).

*PB2 polymerase protein.* The PB2 gene also remained similar to the vaccine strain. The PB2 subunit of polymerase complex is important to host adaptation [50]. A number of amino-acid substitutions (R54K, M66I, D195N, R293K, V344M, I354L, S453T, and V731I) differed from the vaccine strain (Figure 11). The V344M and I354L replacements in PB2, together with the N321K substitution in PA, are the part of the strategy of the swine-origin influenza virus adaptation to the host human population, causing a transition from the severe epidemic to the mild epidemic mode. The V344M and I354L substitutions can modulate the activity of PB2 in its cap-snatching from host mRNA [6,49,51]. Ukrainian isolates grouped with isolates similar to other countries, by acquiring the R299K substitution. Interestingly, viruses divided into groups similar to the HA gene phylogenetic tree (Figure 2), where a small subgroup of isolates from Odessa, Zaporizza, and Singapore had the S453P substitution, with all other isolates having the S453T substitution (Figure 11). It is not known whether this segregation of HA and PB2 has functional consequences, or is indicative of multiple independent introductions of the A(H1N1)pdm09 influenza virus into Ukraine in 2015/16.

**Figure 12:**
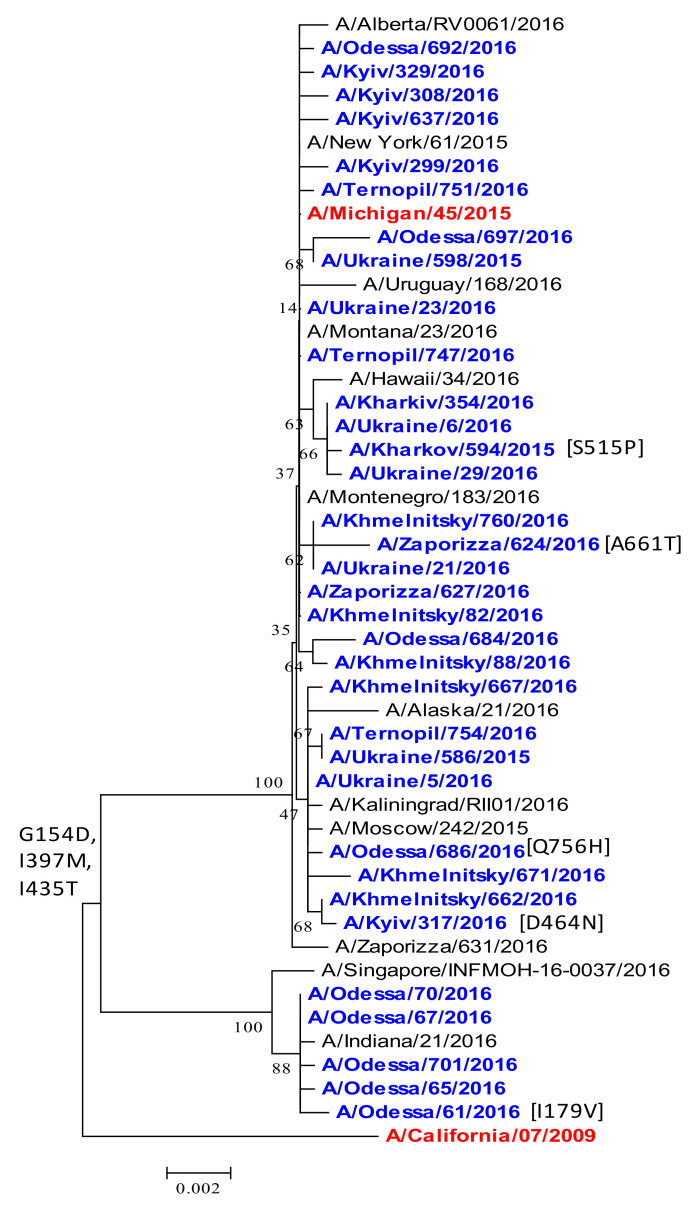
Phylogenetic comparison of A(H1N1)pdm09 influenza virus PB1 from the 2015/16 season. PB1 nucleotide sequences were analyzed by NJ method, Kimura 2-parameter model, with 1000 bootstrap replications. The Ukrainian 2015/16 isolates (blue), reference strains (black), and the vaccine strain (red/bold).

*PB1 polymerase protein.* The PB1 protein of the influenza viruses is responsible for binding to the viral RNA promoter and catalyzing RNA polymerization. Currently, the PB1 subunit structure is the least studied one among subunits of the polymerase complex. PB1 is a RNA-dependent RNA polymerase, which provides a synthesis of viral mRNA and genomic RNA together with a PB2 cap-binding subunit and PA endonuclease subunit [48]. The PB1 phylogenetic tree resembled the PB2 and PA genes. PB1 acquired G154D, I397M, and I435T compared to the A/California/07/2009 vaccine strain (Figure 12). The role of these substitutions is unclear. Some viruses acquired unique single amino-acid substitutions: A/Kharkov/594/2016 had S515P; A/Zaporizzia/624/2016 had A661T; A/Odessa/686/2016 had Q756H; and the A/Kyiv/317/2016 had the D464N substitution. I179V also emerged in the A/Odessa/61/2016 isolate.

## 3. Discussion

The 2015/16 influenza season in Ukraine was particularly severe, dominated by A/(H1N1)pdm09 viruses with an increased prevalence in comparison to the two previous influenza seasons (Figure 1). To understand the nature of the epidemic in Ukraine, we conducted a genetic analysis of A(H1N1)pdm09 viruses reported in clinical cases of ILI from four sentinel clinics in Ukraine in the 2015/16 influenza season. Overall, we found 92% genetic similarity of the pandemic H1N1 influenza viruses in Ukraine to the A/California/07/2009 vaccine strain. Our sequence analysis of A(H1N1)pdm09 in Ukraine supports an epidemiological model of widespread infection (Figure 1), with multiple regional outbreaks and intensive influenza clinical cases [11,16,38]. While we were not able to access detailed patient clinical data (clinical characteristics and comorbidities) or outcomes of individual patients for this study, the sequences analyzed were derived from SARI patients (according to the WHO definition), representing important cases of influenza A(H1N1)pdm09 in Ukraine. We found a wide diversity of A(H1N1)pdm09 strain variants in concurrent circulation, mainly within antigenic drift clades 6B.1 and 6B.2. Clade 6B.1 and 6B.2 A(H1N1)pdm09 viruses became dominant in 2015/16 across the world, including Europe, and were associated with increased hospital admissions and more severe disease in clinical cases [8,10,19,28,38]. The 6B.1 clade dominated human cases in the 2015/16 influenza season in Europe, a pattern we observed by the sequence analysis of Ukrainian A(H1N1)pdm09 strains (Figure 2). In Taiwan, influenza cases in seasons 2013/14 and 2015/16, dominated by 6B, 6B.1, and 6B.2 clades, were at a significantly higher risk of pneumonia (1.8-fold higher risk), the need for oxygen therapy (2.6-fold), and acute respiratory distress syndrome (ARDS, 5.5-fold) [7]; clade 6B.2 infections were still at even a higher risk (3.3-fold) for pneumonia [9,10]. Epidemiological and genetic analyses suggested that clades 6B.1 and 6B.2 harbor some capacity to evade vaccine-induced immunity, mediated by an escape mutations (e.g., S162N, K163Q) in the Sa antigenic site in H1 HA that may evade neutralizing antibodies induced by vaccination [7,8,32].

As contemporaneous vaccinations in Ukraine are typically <5% [11], the potential concern with antigenic variation in influenza A and B strains in the country is that novel strains may evade naturally acquired immunity from previous infection. Thus, we conducted a detailed phylogenetic analysis to understand the evolutionary diversity of A(H1N1)pdm09 viruses in Ukraine in the 2015/16 season. Given the continual genetic drift of influenza A viruses, A(H1N1)pdm09 viruses in Ukraine acquired a number of amino-acid substitutions in each of the 10 principal viral proteins, several that are associated with increased virulence and antigenic drift.

The World Health Organization (WHO) encourages National Influenza Centers (NICs) to conduct ongoing influenza virologic surveillance, to monitor the spread of viruses and their continuous evolution. Combining data from phylogenetic and molecular analyses of influenza viruses is essential to detect virus variants that have undergone antigenic drift, variants with enhanced virulence, or variants with reduced sensitivity to antivirals. Combined genetic, antigenic, and phylogenetic analyses provide improvements in the process of vaccine virus selection and inform patient treatment regimens [28,32,52].

As expected, most of the diversity occurred in the HA and NA genes [18], with the division into two H1 phylogenetic groups, 6B.1 and 6B.2. We found additional substitutions in H1 antigenic sites that emerged in the 2015/16 season and were not detected previously in Ukraine. Antigenic sites consist of the most variable amino acids in the HA molecule of the seasonal human H1N1 viruses that were subjected to intense antibody-mediated immune pressure, the basis for frequent updates to the composition of seasonal influenza vaccines [52]. S84N and the S162N substitution in the Sa antigenic site were observed in all isolates of the 6B.1 genetic group, adding potential N-linked glycosylation sites and an evolutionary advantage to 6B.1 strains that became predominant worldwide beginning in 2015 [10,32]. An additional mutation (S83P) in antigenic site Cb, a receptor-binding residue and target for the neutralizing antibody, was found in 11 of the Ukrainian 6B.1 strains, suggesting continual selective pressure on HA in an immunologically experienced population [32,33,34,52]. Two other substitutions were found in the Ca2 (A141T) and Ca1 (S236P) antigenic sites, in three 6B.1 isolates (Figure 4). Two other A(H1N1)pdm09 strains reported in Ukraine in the 2015/16 season, A/Dnipro/580/2016 and A/Ukraine/7182/2016, contained mutations D222G and D222N in HA, respectively, that are associated with severe disease outcome in patients [16,29,30,31]. Mutations at D222 to Gly (G) or Asn (N) were reported to increase binding to α-2,3 sialic acid in lower respiratory tissues, leading to increased severity of influenza-associated pneumonia [29,31]. This mutation can arise in vivo in mice, and has emerged repeatedly in human epidemics. In one study, in the 2017/2018 influenza season, 32% of severe/lethal A(H1N1)pdm09 cases in Russia contained the D222G/N polymorphism [38].

Variations in other viral proteins were also observed. All 6B-lineage NS1 proteins contained a spectrum of variations (E55K, L90I, I123V, K131E, and N205S) associated with host interferon inhibition and increased virulence in the A(H1N1)pdm09 virus in human and animal models [35,36,37]. In a cluster of Ukrainian isolates, potentially interesting new mutations were found in NS1, namely D2E and N48S in the RNA binding domain and E125D in the NS1 effector domain (Figure 8), that may be associated with NS1 variations reported in severe clinical cases in Russia [38]. N48S co-occurred in three Ukrainian strains with M124T, both of which are gain-of-function mutations that enhance the translation of viral mRNA, possibly by inhibiting antiviral proteins PKR and RAP55 [6,39,40].

Other substitutions were found in NA, NP, M1, M2, NS2, and the polymerase complex among Ukrainian strains, but their effect(s) on the viral life cycle and clinical significance requires further study [38,48,49,50]. NA exhibited a diversity of amino acid mutations; however, no resistance to the antiviral drugs oseltamivir (H275Y) and zanamivir (Q136K) was observed [53]. In the M1 protein, a Q208K substitution differentiated 2015/16 Ukrainian isolates from the previous season; Q208K resides in an alpha-helix determinant of budding and filamentous virion morphology commonly observed when a novel influenza A virus jumps species [45]. All of the A(H1N1)pdm09 isolates analyzed in this study showed the M2 mutation D21G; therefore, all of the isolates most likely had resistance to amantadine [38,46]. As a result, amantadine can no longer be administered as a therapeutic drug against the A(H1N1)pdm09 virus infection in Ukraine. Mutation N321K in PA occurred in all isolates and confers increased virulence [48]. The NP mutation M105T is in a motif under selective pressure (a.a. 98–105) that may affect sensitivity to antiviral protein MXA, and was found in 86% of strains in subsequent influenza seasons [28,38,47].

The observed rapid spread of influenza A(H1N1)pdm09 viruses may be in part explained by antigenic changes in HA, possibly reflecting an escape from immune responses, increased transmissibility, or a change in virulence. Several mutations observed sporadically in Ukrainian A(H1N1)pdm09 influenza viruses in the 2015/16 season became much more prevalent in subsequent years in other countries, suggesting a fitness advantage and ongoing evolutionary pressure [32]. For example, S183P in HA RBD observed in Ukraine isolate A/Zaporizzia/631/2016 increases its affinity for lower respiratory tract α-2,3 SA and accounted for 28% of A(H1N1)pdm09 isolates in 2017/18 [27]. Similarly, in Ukraine in 2016, D2E and E125D in NS1 were also increasingly observed in 6B.1 strains in GISAID, for example, in China from 2016 to 2018. It was suggested that a number of mutations in combination can lead to increased virulence of the A(H1N1)pdm09 influenza viruses, as shown in experiments in animal models [54]. Transmissibility and virulence are dependent on host immune responses and susceptibility, illustrating the complexity of using phylogenetic analyses of virus genomes as a predictor of the severity of an influenza epidemic season. However, recent advances in antigenic cartography to map the landscape of human serological responses to influenza [3], mutational antigenic profiling [32], and next-generation and nanopore sequencing hold promise for the annual sequencing of a large set of whole genomes of influenza A viruses from different geographic regions. Rapidly available sequence data have proven especially important in the epidemic tracing of the SARS-CoV-2 pandemic (2019–2020), and advancing the analysis of virus genetics on the severity of the COVID-19 respiratory disease, providing a model for the tracking and prediction of influenza A and B virus evolution and the selection of influenza vaccine strain candidates can move in a more evidence-based realm [5].

## 4. Materials and Methods

### 4.1. Sample Collection and Diagnostics

Nasal swabs and sputum samples were collected during the routine diagnosis of influenza-like illness (ILI) in patients from ten sentinel hospitals and eight outpatient clinics in four cities of Ukraine (Kyiv, Dnipro, Odessa, and Khmelnytsky). We randomly selected 1246 specimens for PCR diagnostics and sequencing analyses from 4964 hospitalized patients identified by the WHO SARI case definition guidance, including all conditions: acute respiratory infection (ARI) with an onset during the previous 7 days requiring overnight hospitalization; history of fever >38 °C; cough; and shortness of breath or difficulty breathing. All clinical samples were collected under bioethical guidelines for human subjects research under Institutional Review Board approval #281 (11/01/2002), approving authority: Ministry of Health of Ukraine. Clinical samples were analyzed by reverse transcription polymerase chain reaction (RT-PCR) for the influenza A matrix gene (MP) segment and for the influenza type (A, B) and subtype (A/H1N1, A/H3N2) [55]. Clinical samples that were positive for influenza A/H1N1 were cultured on Madin–Darby Canine Kidney (MDCK) cell cultures using standard tissue culture conditions (i.e., 2–4 days of MDCK culture in 10% FBS (Fetal Bovine Serum) medium, gentamycin (50 mg/mL), at 37 °C, 5% CO_2_) [56]. Viruses were sequenced by the World Influenza Center in London using Illumina HiSeq (RNA-SEQ) technology.

### 4.2. Phylogenetic Analysis

For the phylogenetic analysis, sequences of entire genomes of pandemic A(H1N1)pdm09 influenza viruses were obtained from the Global Initiative on Sharing Avian Influenza Data (GISAID; http://platform.gisaid.org/) database. Reference strains included vaccine strain A/California/07/2009 for A(H1N1)pdm09, and isolates from around the world that were temporally and geographically most similar to viruses isolated in Ukraine were included in the phylogenetic analysis. The nucleotide sequences were translated into protein sequences. In the 2015/16 season in Ukraine, we isolated the A(H1N1)pdm09 influenza viruses. The analysis of genetic changes of pandemic viruses was carried out for all of the genes encoding the two surface proteins; hemagglutinin (HA) and neuraminidase (NA); polymerase complex proteins (PA, PB1, PB2); nucleoprotein (NP); M1 and M2 matrix proteins; and non-structural proteins such as NS1 and NS2/NEP (nuclear export protein). The phylogenetic analysis and analysis of mutations were performed using the MEGA7 program by the NJ (neighbor-joining) method and Kimura 2-parameter model, with 1000 bootstrap iterations [57].

### 4.3. Protein Structural Modeling

In order to build a structural model, the mutations, which were found, were put on the protein structures of the A(H1N1)pdm09 influenza virus that was obtained from the Protein Data Bank. The Chimera 1.11.2rc software was used to visualize mutations [58].

## Figures and Tables

**Table 1:** Selected key functional amino-acid substitutions in A(H1N1)pdm09 strains, 2015/16, Ukraine.

Viral Protein	Substitutions	A(H1N1)pdm09 Strains	Potential Functions	Epidemiology	Refs.
**HA**	P83S, I321V, S203T, D97N, S185T, E47K, S124N	Defining group 6B.1, 6B.2 mutations in H1 HA in all Ukrainian strains reported herein.	Antigenic drift; resistance to neutralizing antibody generated by vaccine; S185T RBD mutation may impact receptor specificity.	Group 6B.1, 6B.2 dominated in many countries (2015/16 and 2017/18). Vaccine mismatch.	[7,8,9,10]
	S84N, I216T, S162N, D127E	All group 6B.1 strains	Signature mutations in group 6B.1. Potential novel glycosylation in Sa antigenic site: S162N.	S84N appeared early 2015, a year prior to 6B.1 wave, in India.	[19,20,21]
	F88L	A/Odessa/68/2016 (6B.2)	F88L is on trimeric interaction surface in HA2.		[22]
	T270A	A/Khmelnitsky/663/2016 (6B.1)	Functional significance unknown.	T270A found in 2 strains in swine, Chile (2015).	[23]
	R45K	A/Odessa/685/2016	Antigenic epitope C.	R45K in strains circulating in Kenya in 2015, 2018.	[24]
	N38D, K40N	A/Khelmitsky/81/2016	N38 highly conserved glycosylation site in H1 HA that prevents nAb binding. Loss possibly compensated by K40N.		[25]
	P297S	A/Kharkiv/348/2016	P297S possibly stabilizes HA1 in context of the D222G/E mutation that increases virulence.		[26]
	S183P, S326P	A/Zaporizzia/631/2016	S183P, S326P in H1 RBD; increases binding affinity to a-2,6 SA; S326P in Ca antigenic site.	S183P, S326P strongly selected for by 2018.	[27]
	I91V	A/Khmelnitsky/663/2016, A/Ukraine/25/2016, A/Ternopil/754/2016	I91V is in HA2 domain (functional significance unknown).	I91V found in subset of strains in Bulgaria 2015/16.	[28]
	D222G, D222N	A/Dnipro/580/2016, A/Ukraine/7182/2016	D222G/N increases bindiE17atory tract, increases severity of ILI; increased virulence in mice.	D222G/M mutations occurred in 32% of severe/lethal cases in Russia (2017/18) and elsewhere.	[16,29,30,31]
	A141T	A/Khmelnitsky/727/2016, A/Khmelnitsky/760/2016, A/Zaporizza/631/2016	A141T mutation in Ca antigenic site in HA1.	A141T found in subset of strains in Bulgaria 2015/16.	[28]
	S83P	11 Ukrainian strains (6B.1) *	S83P mutation in Cb antigenic site, epitope E nAb site.	S83P is a common mutation observed in H1N1 isolates and back transferred into swine.	[32,33,34]
**NS1**	E55K, L90I, I123V, K131E, N205S	All Ukrainian strains **	Mutations inhibit host interferon response and gene expression.	Common to NS1 in group 6B.1.	[35,36,37]
	D2E, E125D	17 Ukrainian strains **	D2E in RNA binding domain; E125D in effector domain (functional significance unknown).	Observed in Russia (2015/16) and China (2016–18).	[38]
	N48S, M124T	A/Ukraine/586/2015, A/Ukraine/5/2016, A/Ukraine75/2016	N48S in RNA binding domain enhances viral mRNA translation; M124T inhibits antiviral PKR/RAP55 binding.	N48S present in H5 HPAI NS1 and swine H1 strains in China.	[6,39,40,41]
	I18V	A/Odessa/61/2016	I18V in NS1 site of genetic instability, arises in MDCK passage.	Revertant to swine triple reassortant lineage residue, observed in China.	[41]
	V129I, I182V	A/Dnipro/439/2015	Mutations in NS1 efector domain (functional significance unknown).	Sporadic occurrence, observed in Korea (2013).	[42]
**NA**	H275Y	Not detected	Oseltamivir resistance; reduced fitness.	<2% prevalence in A(H1N1)pdm09 strains.	[43]
	V453M	A/Khmelnitsky/89/2016	V453 is a potentially stabilizing mutations that can co-occur with H275Y.	Sporadic co-occurrence with H275Y.	[43,44]
**M1**	V80I	All Ukrainian strains.	Increased replication in cell culture.	Worldwide.	[5]
	Q208K	62.5% of Ukrainian isolates sequenced in 2015/16. ***	M1 residues 207–209 (alpha helix #12) determine filmentous morphology and budding.	First occurred in Ukraine 2013, 2015, fixed by 2015/16; found in Russia and Bulgaria.	[28,38,45]
**M2**	D21G	All Ukrainian strains.	Resistance to amantadine.	Worldwide.	[38,46]
**NP**	M105T	All Ukrainian strains.	Residues 98–105 form variable motif in human influenza that affects sensitivity/resistance to antiviral protein MXA.	86% of strains in Russia in subsequent year; worldwide.	[28,38,47]
**PA**	N321K	All Ukrainian strains.	Increased virulence.	All A(H1N1)pdm09 strains.	[48]

RBD, receptor binding domain; nAb, neutralizing antibody; SA, sialic acid glycans; ILI, influenza like illness; HPAI, highly pathogenic avian influenza. Refer to strain names on phylogenetic tree in * Figure 2, ** Figure 9, or *** Figure 6. Reference numbers indicated. All strains studied are listed in Table S1.

## Data Availability

All GISAID sequences described in Table S1 are available at: https://www.gisaid.org/.

## Supplementary Materials

The following are available online at https://www.mdpi.com/article/10.3390/v13112125/s1, Table S1: Influenza A(H1N1)pdm09 strains from Ukraine in the 2015-2016 epidemic season.
