# Supplementary material for: Genotypic Variants of Pandemic H1N1 Influenza A Viruses Isolated from Severe Acute Respiratory Infections in Ukraine during the 2015/16 Influenza Season
Source: Viruses. 2021 Oct 21;13(11):2125. doi: 10.3390/v13112125 (PMC8619959; doi:10.3390/v13112125)
Supplement: Supplementary file 1 [file viruses-13-02125-s001.zip › viruses-1377180-supplementary.pdf]

Table S1: Influenza A(H1N1)pdm09 strains from Ukraine in the 2015-2016 epidemic season.

Number	Isolate ID (GISAID)	Strain Name	Collection Date
1	EPI_ISL_192042	A/Ukraine/414/2015	2015-04-16
2	EPI_ISL_192043	A/Ukraine/428/2015	2015-04-13
3	EPI_ISL_192044	A/Ukraine/404/2015	2015-03-03
4	EPI_ISL_192046	A/Ukraine/422/2015	2015-04-15
5	EPI_ISL_192045	A/Dnipropetrovsk/437/2015	2015-04-10
6	EPI_ISL_192051	A/Dnipropetrovsk/443/2015	2015-04-17
7	EPI_ISL_192052	A/Dnipropetrovsk/449/2015	2015-04-27
8	EPI_ISL_192064	A/Dnipropetrovsk/438/2015	2015-04-10
9	EPI_ISL_192067	A/Dnipropetrovsk/439/2015	2015-04-07
10	EPI_ISL_192071	A/Ukraine/368/2015	2015-03-20
11	EPI_ISL_192072	A/Ukraine/371/2015	2015-03-28
12	EPI_ISL_192073	A/Odessa/391/2015	2015-02-25
13	EPI_ISL_192074	A/Ukraine/434/2015	2015-03-23
14	EPI_ISL_192110	A/Ukraine/161/2015	2015-03-02
15	EPI_ISL_192154	A/Ukraine/171/2015	2-15-03-03
16	EPI_ISL_192113	A/Khmelnitsky/462/2015	2015-04-23
17	EPI_ISL_192141	A/Khmelnitsky/461/2015	2015-04-21
18	EPI_ISL_192126	A/Dnipropetrovsk/440/2015	2015-05-06
19	EPI_ISL_214944	A/Ukraine/21/2016	2016-01-03
20	EPI_ISL_216199	A/Ukraine/5/2016	2015-12-29
21	EPI_ISL_216200	A/Ukraine/6/2016	2015-12-29
22	EPI_ISL_216201	A/Ukraine/13/2016	2015-12-30
23	EPI_ISL_216202	A/Ukraine/32/2016	2015-12-28
24	EPI_ISL_216203	A/Odessa/61/2016	2015-12-17
25	EPI_ISL_216204	A/Odessa/63/2016	2015-12-18
26	EPI_ISL_216205	A/Odessa/65/2016	2015-12-15
27	EPI_ISL_216206	A/Odessa/67/2016	2015-12-16
28	EPI_ISL_216207	A/Odessa/68/2016	2015-12-10
29	EPI_ISL_216208	A/Odessa/70/2016	2015-11-27
30	EPI_ISL_216209	A/Ukraine/75/2016	2016-01-06
31	EPI_ISL_216210	A/Khmelnitsky/79/2016	2015-12-15
32	EPI_ISL_216211	A/Khmelnitsky/82/2016	2015-12-24
33	EPI_ISL_216212	A/Khmelnitsky/83/2016	2015-12-25
34	EPI_ISL_216213	A/Khmelnitsky/84/2016	2015-12-25
35	EPI_ISL_216214	A/Khmelnitsky/86/2016	2015-12-25
36	EPI_ISL_216215	A/Khmelnitsky/88/2016	2015-12-23
37	EPI_ISL_216216	A/Ukraine/586/2015	2015-12-11
38	EPI_ISL_216217	A/Ukraine/598/2015	2015-12-17
39	EPI_ISL_216218	A/Kharkov/594/2015	2015-12-10
40	EPI_ISL_218183	A/Ukraine/23/2016	2015-12-21
41	EPI_ISL_218184	A/Ukraine/29/2016	2015-12-25
42	EPI_ISL_230433	A/Kyiv/299/2016	2016-02-02
43	EPI_ISL_230434	A/Kyiv/308/2016	2016-02-03
44	EPI_ISL_230435	A/Kyiv/317/2016	2016-02-03
45	EPI_ISL_230436	A/Kyiv/323/2016	2016-02-02
46	EPI_ISL_230437	A/Kyiv/329/2016	2016-02-02

47	EPI_ISL_230438	A/Kharkiv/344/2016	2016-01-08
48	EPI_ISL_230439	A/Kharkiv/354/2016	2016-01-14
49	EPI_ISL_230440	A/Zaporizza/624/2016	2016-03-03
50	EPI_ISL_230441	A/Zaporizza/627/2016	2016-02-19
51	EPI_ISL_230442	A/Zaporizza/631/2016	2016-02-21
52	EPI_ISL_230443	A/Kyiv/637/2016	2016-03-20
53	EPI_ISL_230444	A/Khmelnitsky/662/2016	2016-02-27
54	EPI_ISL_230445	A/Khmelnitsky/667/2016	2016-02-03
55	EPI_ISL_230446	A/Khmelnitsky/671/2016	2016-03-14
56	EPI_ISL_230448	A/Odessa/679/2016	2016-01-25
57	EPI_ISL_230450	A/Odessa/684/2016	2016-01-29
58	EPI_ISL_230452	A/Odessa/686/2016	2016-02-04
59	EPI_ISL_230454	A/Odessa/688/2016	2016-02-05
60	EPI_ISL_230456	A/Odessa/691/2016	2016-02-06
61	EPI_ISL_230458	A/Odessa/692/2016	2016-02-01
62	EPI_ISL_230462	A/Odessa/697/2016	2016-02-21
63	EPI_ISL_230464	A/Odessa/701/2016	2016-02-27
64	EPI_ISL_230466	A/Khmelnitsky/720/2016	2016-03-20
65	EPI_ISL_230468	A/Khmelnitsky/727/2016	2016-03-22
66	EPI_ISL_230469	A/Ternopil/747/2016	2016-02-22
67	EPI_ISL_230472	A/Ternopil/751/2016	2016-03-04
68	EPI_ISL_230474	A/Ternopil/754/2016	2016-03-01
69	EPI_ISL_230475	A/Dnipro/762/2016	2016-03-16
70	EPI_ISL_230476	A/Khmelnitsky/760/2016	2016-04-20
71	EPI_ISL_230477	A/Dnipro/765/2016	2016-04-01
72	EPI_ISL_248805	A/Dnipro/580/2016	2016-02-05
73	EPI_ISL_ 224674	A/Ukraine/7182/2016	2016-01-22.
